# Unraveling elements of value-based pricing from a pharmaceutical industry’s perspective: a scoping review

**DOI:** 10.3389/fphar.2024.1298923

**Published:** 2024-06-24

**Authors:** Aniek Dane, Carin Uyl-de Groot, Hugo van der Kuy

**Affiliations:** ^1^ Department of Hospital Pharmacy, Erasmus MC, Rotterdam, Netherlands; ^2^ Erasmus School of Health Policy and Management, Erasmus University Rotterdam, Rotterdam, Netherlands

**Keywords:** drug pricing, health economics, health policy, innovative drugs, pharmaceutical industry, pharmacoeconomics, value-based pricing (VBP)

## Abstract

Health authorities use value-based pricing models to determine the value of innovative drugs and to establish a price. Pharmaceutical companies prefer value-based pricing over cost-based pricing. It is ambiguous whether value-based pricing has the same meaning to these stakeholders. We aimed to identify the elements that attribute to value-based pricing of innovative drugs from a pharmaceutical industry’s perspective and as possible starting point for (value-based) contracting of drugs. We performed a scoping review of publications available in scientific databases with terms such as ‘value-based pricing’, ‘pharmacoeconomics’, ‘drug cost’, ‘innovative drug’ and ‘drug therapy’. We included 31 publications, covering value elements of innovative drugs from a pharmaceutical industry’s perspective. Overall, all found elements of value-based pricing were congruent with the elements of value-based pricing from a health authority’s perspective. However, the emphasis placed on the elements differed. The most frequently mentioned elements in our review were economic considerations and cost aspects. Least mentioned were elements regarding cost-effectiveness, disease characteristics and patient characteristics. Although all elements in the drug value framework were present which indicate congruity, there seems controversy on the importance of cost-effectiveness as an element of value. Consequently, establishing a coherent and to all stakeholders’ acceptable framework to value and price innovative drugs seems complicated. Mutual understanding can be found in the value elements societal considerations and healthcare process benefits. Our results supported the importance of economic and cost aspects regarding determination of prices of innovative drugs. Further research is required to quantify the weights of all relevant elements in the drug value framework, observe their possible interlinkages, and to weigh them over time.

## 1 Introduction

As costs of pharmaceuticals keep rising, policymakers, legislators, healthcare professionals, health insurance companies and patients expect pharmaceutical companies to clarify their pricing regimes. However, pharmaceutical companies seem reluctant to disclose their pricing strategies and their ways of determining launch prices of drugs brought to market (Simoens, 2011; Wahlster et al., 2014; Vogler et al., 2017; UCL Institute for Innovation and Public Purpose, 2018; European Commission, 2020; Neumann et al., 2021). This need for transparency is increasing as a growing burden is placed on healthcare systems to ensure sustainable access to healthcare for all patients while budgets are limited (Simoens, 2011; Vogler and Paterson, 2017). Moreover, these prices serve as starting points for price negotiations, contracting and reimbursement decisions later in the process. This is particularly the case for innovative drugs, defined as a completely or partially new active substance or biological entity, or (a) combination of such entities, acting against a disease, relieving symptoms, or preventing a disease through pharmacological or molecular mechanisms, and developed and made available as a medicinal product that can improve the quality of patient management and outcomes (Erice Group, 2008).

In the cases of Kalydeco^®^ and Orkambi^®^, drugs for the treatment of cystic fibrosis, health insurance systems are faced with significant reimbursement challenges upon market entry (Hollis, 2019). The same goes for the reimbursement of Zolgensma^®^, a gene therapy for spinal muscular atrophy, which was heavily debated in the Netherlands (National Health Care Institute, 2021). This drug, which is considered the most expensive drug up to date (Nuijten, 2022), is priced $2.1 million per (one-time) treatment.

Generally, pharmaceutical companies state that prices cannot be calculated by means of a simple equation of several cost aspects, multiplied by a profit margin, the so-called cost-based pricing method (Gregson et al., 2005b). Particularly research & development (R&D) costs seem difficult to attribute to a specific drug, and cost of failures in R&D–promising medicines that eventually do not reach the market–have to be discounted in prices of drugs that do reach the market (DiMasi, 2018). Because of this complexity, pharmaceutical companies prefer to focus on the value of a drug instead of its costs (Gregson et al., 2005b; UCL Institute for Innovation and Public Purpose, 2018). The question arises what value is and how to translate this to pricing methods.

Since 2013, starting with the taxonomy of value-based pricing of drugs by Sussex et al. upon request of the British government, policymakers have assumed that the price of a drug can be considered a function of the perception of its value to patients and society (Towse and Barnsley, 2013a; Sussex et al., 2013). Moreover, the World Health Organization’s (WHO) Collaborating Centre for Pharmaceutical Pricing and Reimbursement Information has defined value-based pricing as ‘setting a price of a new medicine and/or decide on reimbursement based on the therapeutic value a medicine offers, usually assessed through several health technology assessments (HTA) or economic evaluations, which differ by country (WHO Collaborating centre Pharmaceutical Pricing and Reimbursement Information, 2016; Tafuri et al., 2022). However, the value of innovative drugs is a largely unmeasured and misunderstood term (Petrou, 2017). As Petrou described, a definition of real value which is accentuated by superior and significant results in hard and clinically meaningful endpoints is rare in the pharmaceutical sector (Petrou, 2017). Reimbursement agencies determine the value of innovative drugs based on pharma-economic evaluations such as HTA, but these calculations hardly correspond with the prices proposed by the pharmaceutical industry. Therefore, nowadays, it is seen in the United States that payers and pharmaceutical manufacturers have agreed on value-based purchasing contracts in order to link patient outcome to price, amount or nature of reimbursement (Kannarkat et al., 2020; Swart et al., 2020). Nonetheless, Wise et al. stated that the biopharma’s challenge is that the term ‘value’ might mean different things to different stakeholders: ‘value’ perceived as important by the regulatory agency as a therapeutic for a disease in a child might not be the value that is being sought by the patient’s parent or caregiver. Furthermore, outcomes and endpoints are defined differently by different stakeholders for different clinical scenarios (Wise et al., 2018). A richer evidence base and a more open dialog are needed if society is to become more patient-centered in its authorization of innovative therapies (Wise et al., 2018).

Moreover, although intertwined, value and innovation should not be considered alike, where innovation is just one of the determinants of value (Erice Group, 2008). Innovation could, furthermore, be related to other elements of value, such as contribution to scientific knowledge, public health and patient needs, social and economic needs, and environmental impact. Innovation, however, should be considered to be more general than value and comprehensive and invariant across setting and contexts. (Erice Group, 2008).

Based on their systematic review on pricing of medicines, Van der Gronde et al. concluded that value-based pricing and outcome-based pricing are the most promising long-term developments (Van der Gronde et al., 2017). Moreover, value-based pricing has emerged as a preferred alternative to prices determined to what the market will bear (Kaltenboeck, 2020) or other alternatives such as price referencing (Drummond et al., 1997). Nevertheless, it was argued that value-based pricing is more of an art than science due to lack of standardization of value-based pricing practice (Brooks and Geyer, 2016; Jommi et al., 2020) or deemed not appropriate for innovative drugs such as orphan drugs or gene and cell therapies (Drummond and Towse, 2019).

According to the methodological framework of Gregson et al., the value of a drug V is represented by the reference price R (standard of care) plus or minus the differential value D. However, it is not exactly clear what constitutes D in this equation, except that it is a mixture of clinical, economical, and quality of life improvements (Gregson et al., 2005). Furthermore, several methods exist to assess the value of drugs for decision making, although they differ in mission, scope of activities and methodological approaches (Vogler et al., 2017; Neumann et al., 2018). Specifically for oncology drugs, Uyl-de Groot & Löwenberg developed a pricing model based on cost-based-plus pricing to alter the balance between social and economic entrepreneurship. Their model entails elements such as cost of the drug, R&D costs in relation to number of patients, patent period left and profit margin (Uyl-De Groot and Löwenberg, 2018).

As mentioned, Sussex et al*.* developed a taxonomy of value-based pricing (Sussex et al., 2013), succeeded by drug value frameworks developed by Towse & Barnsley in 2013 (Towse and Barnsley, 2013b) and Paulden et al., in 2015 (Paulden et al., 2015). Furthermore, in 2016, PhRMA (Pharmaceutical Research and Manufacturers of America) has declared 15 principles for value assessment frameworks (PhRMA, 2016). This declaration was primarily a response to the value frameworks that were developed to accommodate policy making and pricing decisions of reimbursement agencies and governments. In 2020, the EFPIA (European Federation of Pharmaceutical Industries and Associates) has presented novel pricing and payment models to improve patient access to innovative drugs (EFPIA, 2020). Five principles were set to shape and guide discussions on these pricing models, whereas one of them was the value principle; a high quality, methodologically and mutually agreed value-based framework. However, neither the PhRMA principles nor the EFPIA value principles clearly reveal which elements should be used to determine the value of innovative drugs within the context of value-based pricing. Hence, systematic data that contribute to transparency of pharmaceutical drug pricing and the way value is determined, remain scarce and incomplete (Prasad et al., 2017) and is mainly focused on revealing costs of R&D (DiMasi et al., 2016). Furthermore, in the case of orphan drugs and new cell and gene therapies the need for new approaches to existing drug value frameworks increases (Coyle et al., 2020; Tafuri et al., 2022). Up to date hardly any coherent data or studies exist regarding the value-based pricing methodology of innovative drugs that is used by the pharmaceutical industry. Meanwhile, in March 2017, the European Parliament has adopted a resolution on European Union options for improving access to medicines, which calls for full transparency on the procedures used to determine prices of medical products (European Parliament, 2017).

In an attempt to resolve the controversy over transparency, we believed that governmental policymakers, reimbursement agencies and pharmaceutical companies together should cooperate and decide on the use of jointly accepted drug value framework. This may be useful when, after entering a country’s market, governmental, health authorities, health insurers and care providers –- depending on the country - start various kinds of HTA and/or cost-effectiveness assessments, managed entry agreements and price negotiations, and reimbursement arrangements as part of the (value-based) contracting process.

From literature, we were acquainted with drug value frameworks from a policymaker’s perspective, but we were unaware what resembled a drug value framework from a pharmaceutical perspective. Therefore, the aim of our study was to identify the pricing elements that attribute to the value of innovative drugs as perceived by the pharmaceutical industry. A scoping review was chosen in order to identify and map key characteristics to the concept of value-based pricing (Munn et al., 2018).

## 2 Methods

### 2.1 Search strategy

We performed a systematic search strategy to collect and analyze elements of value-based pricing. We limited our search to publications in scientific journals to avoid public debates and marketing statements on the subject published in grey literature.

The review was performed in five subsequent steps: 1) identification of publications; 2) screening titles and abstracts; 3) screening full texts; 4) analyzing full texts by means of a value framework, and 5) validation.

First, published studies were identified using the electronic databases Embase, Medline, Web of Science, Econlit and Google Scholar. Searches were performed with terms such as ‘pricing’, ‘pharmacoeconomics’, ‘drug cost’, ‘orphan drug’, ‘drug therapy’, ‘value-based pricing’, ‘pharmaceutical’, ‘innovation’, ‘rare disease’ and ‘medicine’. The complete search strategy is presented in File S1 in the Supplementary Material. The initial search was performed in February 2020 and updated in August 2022 and included all publications from inception to date that matched with the targeted word combinations. No additional filters for language or quality of evidence were applied at this stage. Only duplicate records were excluded from the initial abstract screening.

To execute the second step–screening titles and abstracts–a list of criteria was made to include eligible publications. Publications were included if they met the following criteria: 1) pharmaceutical industry’s perspective; 2) situated in high-income and OECD (Organization for Economic Cooperation and Development) country; 3) mentioning drug pricing, drugs costs and/or value of drugs; 4) describing price elements and/or value elements; 5) studying pricing of innovative prescription drugs and/or orphan drugs.

We choose these particular criteria for the following reasons. Criterion one was selected to only include articles which were written from a pharmaceutical industry’s point of view, since the aim of this study was to identify elements of value-based pricing from this perspective. Criterion two was selected because pricing or value discussions on pharmaceuticals differ between high- and low-income countries (OECD, 2008). Criteria three and four were selected to include articles on pricing and value and to explicitly exclude articles on economic, cost or cost-effectiveness analyses of specific drugs for not being the area of research in this study. Furthermore, we did not distinguish between prices or value of drugs at launch or at a later point in time–e.g., we included both patented drugs and drugs after patent expiry. Finally, criterion five was selected to include articles discussing innovative pharmaceuticals and to exclude pricing of generic pharmaceuticals or over-the-counter (OTC) drugs. Furthermore, Abstracts (A) and summaries (S) were excluded.

The third step consisted of screening full texts. Eligible publications had to be written in English and had to be available for reviewing. Publications were excluded if they did not meet these criteria. In order to analyze the full texts of the included publications in the fourth step, a framework was generated based on elements that all were present in the existing drug value frameworks of Sussex et al., Towse & Barnsley, Paulden et al. and Lakdawalla et al. (Towse and Barnsley, 2013a; Sussex et al., 2013; Paulden et al., 2015; Lakdawalla et al., 2018). From these models we extracted the following elements: health effects (e.g., quality of live, (cost-)effectiveness, outcomes); patient and disease characteristics (e.g., child/adult, unmet need, severity and rarity); societal benefits (e.g., increased labor productivity, health gain on population level); healthcare process related aspects (e.g., convenience in administration, less time-consuming, reduced hospitalizations); innovation (advancement of scientific knowledge achieved by the development of medicines (Sussex and Towse, 2013), future products as a consequence of approval of a product today (Towse and Barnsley, 2013b), scientific spillover; future benefits of current innovations (Lakdawalla et al., 2018)); risks (e.g., uncertainty of outcome, financial risks, legal considerations); costs (e.g., cost of R&D, cost of capital, cost of failure) and economic factors (e.g., business, industrial and commercial considerations).

For structuring and quality and sensitivity analysis of the included articles additional data were collected: first author; year of publication; publication source; type of publication; studied country/countries; research period; objective; medical condition; described name of the drug; composition of the drug; type of drug; involvement of pharmaceutical industry with the publication. While analyzing, relevant text passages of the included publications were copied and pasted into the framework and highlighted for quick recognition. The last step was initiated to minimize the risk of selection bias and to enhance internal validity and consisted of analyzing a random sample of included publications after completion of the framework by the two authors not involved in analyzing full texts of all included publications.

### 2.2 Quality assessment

Overall, to minimize the risk of bias several co-workers were involved. To conduct the literature search and extraction of the eligible publications one of the authors, (AD), was supported by a co-worker of the Erasmus MC Medical Library. Subsequently, all authors, independently, screened the titles and abstracts, thereby looking for publications that met the above-mentioned criteria. Next, one author (AD) screened the full texts of the publications included and then analyzed these publications using the developed framework. Successively, one co-worker of the Erasmus MC Hospital Pharmacy Department independently analyzed the full texts of the included publications. The two separately filed out frameworks were then compared and discussed. Lastly, two authors (CU, HK) each analyzed a random sample of six of the eligible publications of the third round and compared their findings with the completed framework. Differences were resolved via discussion and consensus. To ensure the quality of reporting, the Preferred Reporting Items for the Systematic reviews and Meta-Analyses extension for Scoping Reviews (PRISMA-ScR) checklist was used (Tricco et al., 2018). The completed checklist is available in File S2 of the Supplementary Material. Furthermore, a sensitivity analysis was performed by withdrawing low quality publications such as case reports and conference papers. We did not register or publicly publish the study protocol.

## 3 Results

### 3.1 Literature search results

**FIGURE 1:**
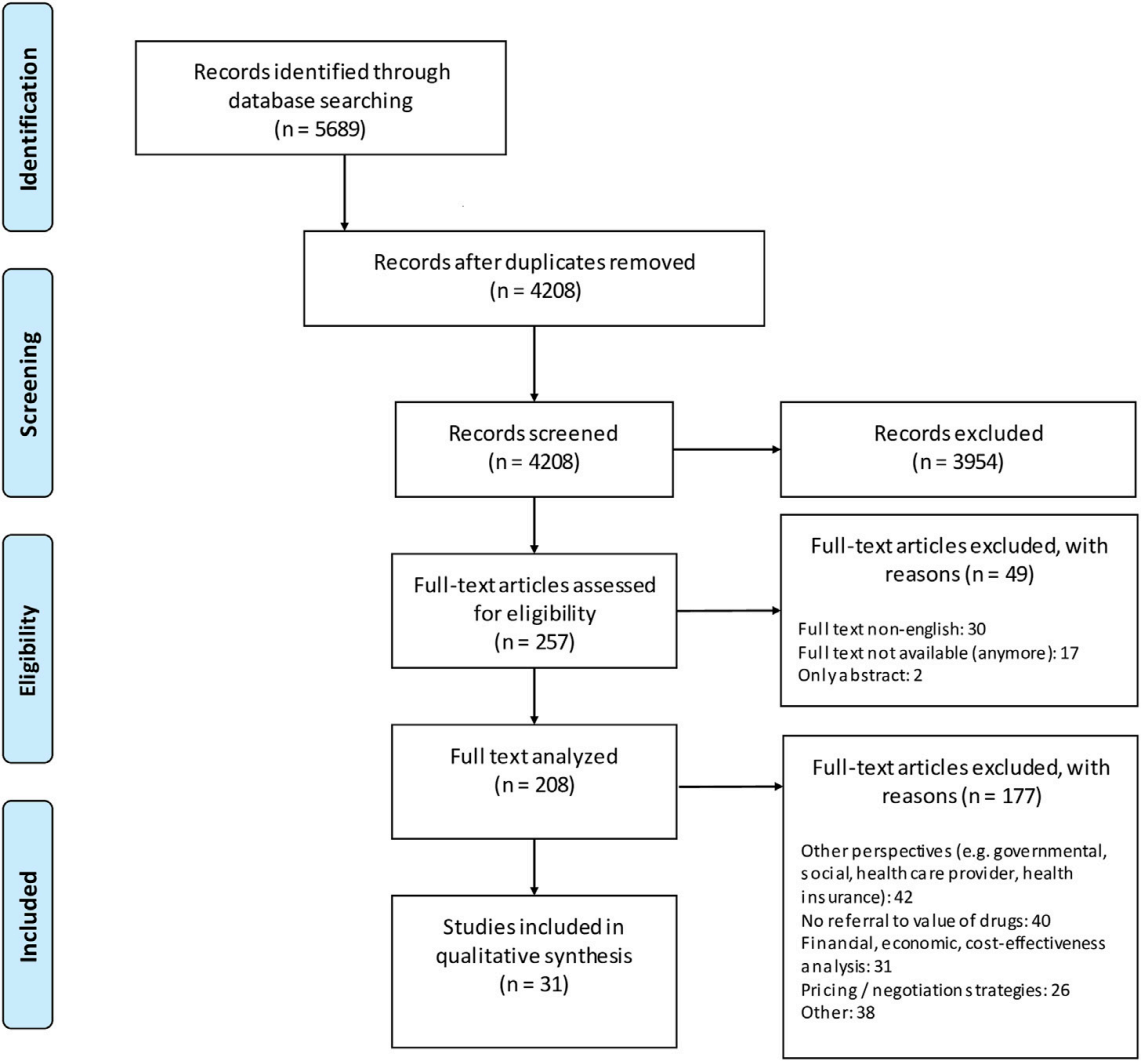
Flow chart.

The database search identified 5,689 unique publications. The final number of publications included in the review was 31. The flow chart in Figure 1 illustrates reasons for exclusion and the number of excluded publications (Moher et al., 2009). All included publications were analyzed for concepts that attributed to the specific value elements and were placed into the framework. Subsequently, when analyzing the concepts in the framework, we identified several sub-elements per element. By grouping the results, we were able to quantify elements and sub-elements and we, thereby, replaced some of the concepts placed in the element ‘other’ to an already defined element, and subsequently, grouped the remaining concepts placed in the element ‘other’ and renamed it ‘drug development complexity’ as displaced in Table 1.

**TABLE 1:** Overview of included publications and results^*^.

General information	Publication year	Studied countries	Publication type	Drug type	Involvement of pharmaceutical industry
	**1990–2000: 5** Siegelman S (1991) Vagelos PR (1991) Weidenbaum ML (1993) Murray MD (1998) Lu ZJ (1998) **2001–2010: 8** Calfee JE (2001) Dockhorn RJ (2005) Ruffolo RR (2005) Sollano J (2008) Tambuyzer E (2010) Lockhart MM (2010) Reinhart R (2010) Zhong X (2010) **2011–2022: 18** Davies JE (2012) Numerof RE (2012) Dickov V (2012) Rollet P (2013) Silverman E (2013) Saadi E (2014) Winegarden W (2014) Kibble A (2015) Gutierrez L (2015) Morrison C (2015) Pauwels K (2016) Patel KR (2017) Wise J (2018) Barkan J (2019) de Sola-Morales O (2019) Coyle D (2020) Garrison LP (2021) Postma MJ (2022)	**USA: 14** Siegelman S; Vagelos PR; Weidenbaum ML; Murray MD; Lu ZJ Calfee JE Dockhorn RJ; Ruffolo RR Sollano J; Silverman E; Winegarden W; Patel KR; Garrison LP **Developed/high income countries: 10** Zhong X; Davies JE; Numerof RE; Dickov V; Saadi E; Morrison C; Wise J; Barkan J; Coyle D; Postma MJ **Europe: 4** Rollet P, Kibble A, Guttierrez L, de Sola-Morales O **US/Europe/Japan: 1** Tambuyzer E **Belgium: 1** Pauwels K **New Sealand:** Lockhart R	**Journal article - review: 14** Vagelos PR; Weidenbaum ML; Murray MD; Dockhorn RJ; Sollano J; Reinhart R; Zhong X; Davies JE Dickov V; Rollet P; Patel KR; Wise J; Coyle D; Postma MJ **Journal article - opinion: 6** Calfee JE; Tambuyzer E; Silverman E; Saadi E; Gutierrez L; Garrison LP **Conference summary: 2** Kibble A, Barkan J **Journal article - qualitative analysis: 2** Lockhart MM, Pauwels K **Journal article - quantitative analysis: 1** Lu ZJ **Journal article - special report: 1** Siegelman S **Journal article - editorial: 1**de Sola-Morales O **Journal article - news: 1** Morrison C **Magazine article - opinion**: 1 Numerof RE **Report**: 1 Winegarden W **Case report**: 1 Ruffolo RR	**Prescription drugs: 13** Siegelman E; Vagelos PR; Weidenbaum ML; Murray MD; Calfee JE; Ruffolo RR; Sollano J; Lockhart MM; Zhong X; Davies JE; Numerof RE; Kibble A; Wise J **Orphan drugs: 10** Dockhorn RJ; Tambuyzer E; Reinhart R; Rollet P; Silverman E; Gutierrez L; Morison C; Patel KR, de Sola-Morales O; Postma MJ **Patented/innovative drugs: 8** Lu ZJ; Dickov V; Saadi E; Winegarden W; Pauwels K; Barkan J; Coyle D; Garrison LP	**Yes: 21** Vagelos PR; Lu ZJ; Calfee JE; Dockhorn J; Ruffolo RR; Sollano J; Tambuyzer E; Reinhart R; Zhong X; Davies JE; Numerof RE; Rollet P; Silverman E; Saadi E; Gutierrez L; Pauwels K; Wise J; de Sola-Morales O; Coyle D; Garrison LP; Postma MJ **No/unknown: 10**> Siegelman S; Weidenbaum ML; Murray MD; Lockhart MM; Dickov V; Winegarden W; Kibble A; Morison C; Patel KR; Barkan J
**Societal considerations: 30**	**Social/unmet needs: 7**	**Quality of life on a population level: 7**	**Increased productivity: 6**	**Impact on healthcare budget: 8**	**Insurance value: 2**
	Vagelos PR; Wise J; de Sola-Morales O; Morrison C; Weidenbaum ML; Saadi E; Gutierrez L	Siegelman S; Vagelos PR; Saadi E; Wise J; Davies JE; Coyle D; Garrison LP	Siegelman S; Vagelos PR; Saadi E; Pauwels K; Wise J; Garrison LP	Pauwels K; de Sola-Morales O; Tambuyzer E; Rollet P; Weidenbaum ML; Coyle D (2^**^); Garrison LP	Coyle D; Postma MJ
**Economic considerations: 42**	**Return on investment: 18**	**Willingness-to-pay: 13**	**Country-specific pricing characteristics: 5**	**Competition: 6**	
	Vagelos PR; Weidenbaum ML; Calfee JE (3); Tambuyzer E (2); Lockhart MM; Zhong X; Rollet P; Numerof RE; Dickov V (2); Winegarden W (2); Murray MD; Pauwels K; Dockhorn RJ	Wise J; Calfee JE; Sollano J; Pauwels K; Morrison C; Tambuyzer E; Zhong X; Numerof RE; Silverman E; Dickov V; Saadi E; de Sola-Morales O; Coyle D	Vagelos PR; Wise J (2); Zhong X; de Sola-Morales O	Calfee JE; Lu ZJ; Vagelos PR; Rollet P; Pauwels K; Sollano J	
**Healthcare process considerations: 31**	**Superior treatment: 14**	**Reduction other costs of healthcare delivery: 10**	**Patient access: 3**	**Preferences: 2**	**Logistics & treatment challenges: 2**
	Vagelos PR (2); Weidenbaum ML; Zhong X; Numerof RE; Dickov V; Silverman E (2); Winegarden W; Gutierrez L; Morrison C Coyle D; Postma MJ; Garrison LP	Siegelman S; Pauwels K; Vagelos PR; Murray MD; Saadi E; Winegarden W; Morrison C; Wise J; Zhong X; Barkan J	Saadi E; Vagelos PR; Gutierrez L	Zhong X (2)	Coyle D; Postma MJ
**Patient characteristics: 9**	**Heterogeneity of patients: 3**	**Knowledge of patient population: 3**	**Personalized medicine: 2**	**Patient’s weight: 1**	
	Zhong X; Gutierrez L; Rollet P	Numerof RE; Gutierrez L; Barkan J	Zhong X; Numerof RE	Morrison C	
**Disease characteristics: 14**	**Disease rarity: 6**	**Type of disease: 3**	**Disease heterogeneity: 3**	**Other treatment options: 1**	**Disease severity: 1**
	Tambuyzer E; Silverman E; Gutierrez L; Rollet P; Barkan J; Davies JE	Lu ZJ; Saadi E; Gutierrez L	Zhong X; Gutierrez L (2)	Lockhart MM	Coyle D
**Effectiveness: 23**	**Outcome: 12**	**Clinical value: 7**	**Cost-effectiveness: 4**		
	Weidenbaum ML; Dockhorn RJ; Dickov V; Morrison C; Murray MD; Winegarden W; Barkan J; Numerof RE; Pauwels K; Zhong X; Garrison LP; Coyle D	Lu ZJ; Zhong X; Numerof RE; Pauwels K; Rollet P; Gutierrez L; Wise J	Morrison C; Calfee JE; Siegelman S; Coyle D		
**Cost aspects: 39**	**R&D costs: 18**	**Cost of failure: 8**	**Manufacturing costs: 5**	**Cost of capital: 4**	**Regulatory & commercialization costs: 4**
	Vagelos PR; Weidenbaum ML; Calfee JE (2); Dockhorn RJ; Sollano J; Tambuyzer E; Lockhart MM (2); Reinhart R; Zhong X; Davies JE; Numerof RE; Winegarden W; Gutierrez L; Patel KR; Rollet P; Coyle D	Winegarden W; Calfee JE; Sollano J; Lockhart MM; Zhong X; Davies JE; Rollet P, Coyle D	Calfee JE; Tambuyzer E; Lockhart MM; Reinhart R; Winegarden W	Rollet P; Tambuyzer E; Vagelos PR; Zhong X	Tambuyzer E (3); Wise J
**Innovational aspects: 17**	**New treatments: 6**	**Future research: 6**	**Innovation n.o.d.: 5**		
	Rollet P; Saadi E; Gutierrez L; Winegarden W; Wise J; Weidenbaum ML	Tambuyzer E; Pauwels K; Zhong X; Numerof RE; de Sola-Morales O; Coyle D	Rollet P; Murray MD; Davies JE; Kibble A; Coyle D		
**Drug development complexity: 24**	**Risks: 14**	**Safety: 5**	**Duration & complexity: 5**		
	Calfee JE; Ruffolo RR, Tambuyzer E (2); Lockhart MM; Zhong X (3); Dickov V; Rollet P; Saadi E (2); Winegarden W; Barkan J	Tambuyzer E; Sollano J; Davies JE; Numerof RE; Dickov V	Tambuyzer E; Zhong X; Dickov V; Saadi E; Gutierrez L		

*For reasons of readability of the table only the first author of the publications included is mentioned. Full disclosure can be found in the reference section.

^a^ The number between brackets is the number of different components (sub-elements) that belong to a specific element.

### 3.2 Societal considerations

Concerning societal considerations–benefits to society –, we grouped the concepts found in literature into five sub-elements. First, seven publications linked value to social or unmet needs (Weidenbaum, 1993; Saadi and White, 2014; Gutierrez et al., 2015; Morrison, 2015), more in detailed described as societies should care for those in need (de Sola-Morales, 2019), or should help to ensure that patients can obtain the medicine they need (Vagelos, 1991; Wise et al., 2018). Secondly, seven publications mentioned quality of life on a population level such as improved population health (Saadi and White, 2014) and population wellbeing (Wise et al., 2018), reduction of morbidity rate (Siegelman, 1991), reducing disability days and potential years of life lost before the age of 65 (Vagelos, 1991), and long-term benefits for humans (Davies et al., 2012) in general and, specifically, for caregivers and family (Coyle et al., 2020; Garrison et al., 2021). Thirdly, six publications mentioned increased productivity (Vagelos, 1991; Saadi and White, 2014; Wise et al., 2018) and recessed absent from work (Siegelman, 1991; Pauwels et al., 2016) and, in the case of cell and gene therapies, even lifetime productivity (Garrison et al., 2021). A fourth sub element was related to the impact on the national healthcare budget and potential cost savings to society (Coyle et al., 2020) and whether prices were seen as justifiable to payers (Rollet et al., 2013) in accordance with national budgets and priorities (Pauwels et al., 2016). Conversely, in two publications it was mentioned that the burden placed on society was low, stating that innovative drugs only have been making up a small proportion of total healthcare expenditures (Weidenbaum, 1993) and because the number of patients treated with these drugs is low (Tambuyzer, 2010). Lastly, in recent publications the value of especially cell & gene therapies was linked to insurance value, which can be distinguished in two types of risk protection on a population level: physical risk protection (reduced fear of a disease) and financial risk protection (covering cost of treatment through an insurance system) (Postma et al., 2022).

### 3.3 Economic considerations

With respect to drug price-related economic considerations, we grouped the concepts into four sub-elements: 1) return on investment; 2) willingness-to-pay; 3) country-specific pricing characteristics and 4) competition. In 18 publications drug prices were linked to return on investment. This was described by: i) an appropriate return on research investment (Vagelos, 1991); ii) the basic incentive to make such investments in the possibility of high profits (Weidenbaum, 1993; Calfee, 2001); iii) the hope of someday obtaining large profits from rare success (Calfee, 2001), and iv) making profits in order to be able to continue to reinvest in the developments of new medicines for complex conditions (Tambuyzer, 2010; Rollet et al., 2013). Furthermore, economics of potential drugs were studied upfront (Dockhorn, 2005). A second sub-element was willingness-to-pay, reflected by 13 publications and stated by, e.g., Wise et al. (Wise et al., 2018) as “the pharmaceutical challenge: [the] therapeutics must meet unmet patient needs at a cost that society can afford”. Moreover, Coyle et al. stated that “the value of innovative therapies should reflect society’s preferences to pay more for greater health gain, health gains for highly debilitating conditions or for survival extension near end-of-life” (Coyle et al., 2020). Furthermore, in one publication it was stated that if societies were willing to pay for value drug prices could differ between different medical indications (Pauwels et al., 2016). Hence, higher value to patients should actually command a higher price (Morrison, 2015). The third sub-element considered country-specific price differences due to price elasticity within a society (Zhong, 2010), variations in government price controls, healthcare financing practices (Vagelos, 1991) or a supportive attitude towards business environment for innovative pharmaceuticals (Wise et al., 2018). Price differences also occurred when prices in one country subsidized prices in another country (de Sola-Morales, 2019) or were due to different outcomes of the value of a drug based on different HTA-technologies and assessment methodologies (Wise et al., 2018). Finally, the fourth sub-element related to competition or lack thereof, in which competition had a dampening effect on drug prices (Lu and Comanor, 1998; Calfee, 2001) and market protection such as market exclusivity or patenting enabled the pharmaceutical industry to recoup costs. Then again, these protection policies were not preventing the marketing of other orphan medicinal products (Rollet et al., 2013). Indeed, companies would anticipate price pressure and price erosion, leading to higher prices at initial price setting (Pauwels et al., 2016). Therefore, the ultimate challenge is to achieve success in the face of shorter patent exclusivity periods and global enforcement of more stringent price controls and reimbursement criteria (Sollano et al., 2008).

### 3.4 Healthcare process considerations

In almost all publications, a drug’s price was related to healthcare process considerations and, specifically, to superior treatment or reduction of other costs of healthcare delivery. Concerning superior treatment, efficacy with comparator products (Zhong, 2010; Silverman, 2013; Gutierrez et al., 2015) and medical and therapeutic advances (Dickov, 2012) were mentioned, especially reduction of surgery (Vagelos, 1991; Weidenbaum, 1993; Winegarden, 2014). Four publications mentioned substitution of a lifetime of medical interventions to a one-time treatment (Morrison, 2015; Coyle et al., 2020; Garrison et al., 2021; Postma et al., 2022) of which three publications were of the last 3 years and specifically regarding cell & gene therapies. According to Coyle *et al.* (Coyle et al., 2020) and Postma et al. (Postma et al., 2022), these therapies, moreover, faced logistic, procedural and treatment challenges for healthcare delivery, including increasing treatment costs. Subsequently, superiority was stated to come at a higher price (Vagelos, 1991; Zhong, 2010). Conversely, the reduction of other healthcare delivery costs, especially reduced hospitalizations, was mentioned in seven publications (Siegelman, 1991; Vagelos, 1991; Murray and Deardorff, 1998; Saadi and White, 2014; Winegarden, 2014; Pauwels et al., 2016; Wise et al., 2018). Three publications mentioned patients’ accessibility to innovative therapies to be an important consideration (Vagelos, 1991; Saadi and White, 2014; Gutierrez et al., 2015). Moreover, prices should be kept at reasonable levels, as new therapies were useless if patients could not access them (Vagelos, 1991). Gutierrez et al. added ethical perspectives, such as the rule of rescue or the equity of opportunity for patients to benefit (Gutierrez et al., 2015). In one publication a relationship was found between drug prices and patients’ and physicians’ preferences (Zhong, 2010).

### 3.5 Patient characteristics

Patient characteristics were mentioned in six publications and were mostly linked to the heterogeneity of the patient population and to the understanding of the patient population (Zhong, 2010; Numerof and Abrams, 2012; Rollet et al., 2013; Gutierrez et al., 2015; Barkan, 2019). Scarcity of the available patient pool and the heterogeneous populations made it difficult to identify validated clinical endpoints (Rollet et al., 2013) and, subsequently, forced pharmaceutical companies to develop a deeper understanding of that population’s characteristics (Numerof and Abrams, 2012) and to contribute to the value of a patient’s hope (Barkan, 2019). The concept of personalized medicine should suggest that many new drugs will only reach a proportion of the patients suffering from a particular disease (Zhong, 2010). In one publication the price of a drug was related to the patient’s weight (Morrison, 2015).

### 3.6 Disease characteristics

Regarding disease characteristics, in 14 publications drug prices were considered to be related to the rarity or severity of the disease and the type of disease. According to several publications, the rarity of the disease was linked to complexity of drug development due to low prevalence (Tambuyzer, 2010; Barkan, 2019), higher unit costs (Davies et al., 2012) and a small number of potential patients (Rollet et al., 2013; Silverman, 2013). Regarding the type of disease, it was mentioned in several publications that drug prices were related to disease heterogeneity (Zhong, 2010), level of knowledge on the disease (Gutierrez et al., 2015), or whether a disease was considered more severe (Gutierrez et al., 2015), more acute (Lu and Comanor, 1998) or was associated with certain perceptions (Saadi and White, 2014), such as inherited diseases or diseases acquired by lifestyle. In one publication, the focus was to find a solution for diseases with insufficient treatment options (Lockhart et al., 2010).

### 3.7 Effectiveness

Effectiveness in relation to drug prices was mentioned in 23 publications whereas half of the publications described effectiveness as outcome effects, such as extending life expectancy (Dickov, 2012), saving lives (Dockhorn, 2005), or in general improving the quality of a patient’s life (Weidenbaum, 1993; Murray and Deardorff, 1998; Dockhorn, 2005; Winegarden, 2014; Morrison, 2015; Pauwels et al., 2016; Barkan, 2019; Coyle et al., 2020). Furthermore, it was stated that actual prices are closely related to a patient’s benefit of the treatment (Weidenbaum, 1993; Winegarden, 2014) or to a patient’s responsiveness to the treatment (Zhong, 2010). In publications on cell & gene therapy price was related to the benefits of a one-time (Garrison et al., 2021) or non-chronic treatment, thereby lowering the number of hospitalizations or chronic care for patients (Coyle et al., 2020). A second element that referred to effectiveness was clinical value, whereas two publications stated a direct relationship between therapeutic improvement and drug price at market introduction (Lu and Comanor, 1998; Zhong, 2010). Cost-effectiveness was mentioned in four publications, indicating a relation between price and cost-effectiveness (Siegelman, 1991; Morrison, 2015; Coyle et al., 2020) yet one publication was opposed to that, stating that costs of drug development and ultimate benefits of that drug are not necessarily related (Calfee, 2001).

### 3.8 Costs

Almost all publications mentioned cost aspects in relation to drug pricing, which were grouped into five sub-elements. First, most publications mentioned cost for R&D, indicating that cost of R&D is a major factor in determining the price of a new drug, including the cost for discovering a new drug (Dockhorn, 2005; Lockhart et al., 2010; Patel, 2017; Coyle et al., 2020). Especially clinical trials place a great burden on R&D costs (Weidenbaum, 1993; Dockhorn, 2005; Lockhart et al., 2010; Tambuyzer, 2010; Davies et al., 2012; Rollet et al., 2013; Winegarden, 2014). Therefore, drug prices should allow for companies to recoup their R&D costs (Reinhart and Modrzjewski, 2010; Zhong, 2010; Gutierrez et al., 2015). Furthermore, in addition to cost of R&D, cost of failures was mentioned (Calfee, 2001; Sollano et al., 2008; Lockhart et al., 2010; Zhong, 2010; Davies et al., 2012; Winegarden, 2014; Coyle et al., 2020). Rollet et al. (Rollet et al., 2013) stated that “the proportion of failures is the most important driver for R&D costs”, whereas Calfee (Calfee, 2001) argued to “bear in mind research failures and bankruptcies that may have proceeded the creation of a financially successful new drug”. Third, cost of production and manufacturing was mentioned in five publications (Calfee, 2001; Lockhart et al., 2010; Reinhart and Modrzjewski, 2010; Tambuyzer, 2010; Winegarden, 2014). Fourth, four publications mentioned cost of capital (Vagelos, 1991; Tambuyzer, 2010; Zhong, 2010; Rollet et al., 2013) and finally, two publications touched upon the regulations and commercialization costs (Tambuyzer, 2010; Wise et al., 2018).

### 3.9 Innovation

Murray and Deardorff stated (Murray and Deardorff, 1998) that “innovation is the lifeblood of the pharmaceutical industry” and it is, therefore, argued that the business models of pharmaceutical companies are associated with high prices to counterbalance the large focus on innovation (Rollet et al., 2013). Making a profit is considered to be an important driver for further research and offers possibilities for investing in future pipelines and tomorrow’s medicines (Tambuyzer, 2010; Rollet et al., 2013; Pauwels et al., 2016; de Sola-Morales, 2019). Moreover, the value of innovation is important because of the scientific spill-over effect: knowledge gained from one drug leads to the development of other valuable innovations (Coyle et al., 2020). Furthermore, limiting prices of drugs could have a negative impact on innovation and could result in not being able to fulfill unmet needs of patients (Kibble & D'Souza, 2015; Weidenbaum, 1993; Winegarden, 2014; Wise et al., 2018).

### 3.10 Drug development complexity

The last element–drug development complexity–was composed of the sub-elements risks, safety, and complexity. Most publications mentioned risks to be included in drug pricing. As Tambuyzer (Tambuyzer, 2010) stated “the price of a drug and the corresponding cost per patient is determined by the risk taken to develop the product, which is reflected in the profit potential”. Drug development was considered to be a high risk industry (Ruffolo, 2005) from several perspectives such as high risk levels of the R&D process and failure rates (Lockhart et al., 2010; Zhong, 2010; Dickov, 2012; Saadi and White, 2014; Barkan, 2019), unique risks reflected by frequent mergers and acquisitions (Zhong, 2010), increase of unprecedented drug withdrawals and product liability lawsuits (Zhong, 2010), structural hurdles contributing to an increased risk of failure (Rollet et al., 2013), and risks inherent to commercial success and adequate return on investment (Saadi and White, 2014; Winegarden, 2014). A second sub-element was safety in which the ultimate challenge is to achieve success in the face of increasing demands, i.e., increasing regulatory hurdles for evidence of safety and efficacy (Sollano et al., 2008; Davies et al., 2012; Numerof and Abrams, 2012). Finally, in five publications it was considered the complexity and duration of the entire process of drug development (Tambuyzer, 2010; Zhong, 2010; Dickov, 2012; Saadi and White, 2014; Gutierrez et al., 2015) to be reflected in the price.

### 3.11 Summary of covered elements

We found nine different elements, and we grouped several sub-elements per element (see Table 2). The order of the elements resembles how often an element was mentioned in the included publications and is, therefore, considered an indication of the hierarchy of the elements. In order of importance: 1) economic considerations; 2) cost aspects; 3) healthcare process considerations 4) societal considerations; 5) drug development complexity; 6) effectiveness; 7) innovation; 8) disease characteristics; and 9) patient characteristics. The sensitivity analysis in which eight articles (25%) were excluded that were not reviews or analyses (i.e., conference summary, special report, editorial, news, magazine article, report, case report) revealed a slight change the elements hierarchy in which healthcare process consideration and societal considerations switched, as well as innovation and disease characteristics: (1) economic considerations; 2) cost aspects; 3) societal considerations; 4) healthcare process considerations 5) drug development complexity; 6) effectiveness; 7) disease characteristics; 8) innovation; and 9) patient characteristics). The patient characteristics element diminished from 9 times to 5 times mentioned (a 55% reduction). Economic and cost considerations remained in the same position (Table 3). Lastly, we analyzed the order of the elements over time. In publications appeared in the period from 1991 to 2000 societal and healthcare process considerations were more prominently present, whereas publications appeared between 2001 and 2010 were more focused on business aspects, such as costs, economic considerations and drug development complexity. From 2011 up to 2022 the focus was on a combination of business and societal aspects. However, throughout the years patient and disease characteristics were not among the top five of considered elements of value (Table 4).

**TABLE 2:** Hierarchy of elements to consider in value-based pricing from a pharmaceutical industry’s perspective.

Economic considerations	Cost aspects	Healthcare process considerations	Societal considerations	Drug development complexity	Effectiveness	Innovational aspects	Disease characteristics	Patient characteristics
Return on investment Willingness-to-pay Country-specific pricing characteristics Competition	R&D costs Cost of failure Manufacturing costs Cost of capital Regulatory & commercialization costs	Superior treatment Reduction other cost of healthcare delivery Patient access Preferences Logistics & treatment challenges	Unmet or social needs Quality of life on population level Increased productivity Impact on healthcare budget Insurance value	Risks Safety Duration & complexity	Outcome Clinical value Cost-effectiveness	New treatments Future research Innovation	Disease rarity Type of disease Disease heterogeneity Other treatment options Disease severity	Patient heterogeneity Knowledge of patient population Personalized medicine

**TABLE 3:** Hierarchy of elements after sensitivity analysis.

Economic considerations	Cost aspects	Societal considerations	Healthcare process considerations	Drug development complexity	Effectiveness	Disease characteristics	Innovational aspects	Patient characteristics
Return on investment Willingness-to-pay Country-specific pricing characteristics Competition	R&D costs Cost of failure Manufacturing costs Cost of capital Regulatory & commercialization costs	Unmet or social needs Quality of life on population level Increased productivity Impact on healthcare budget Insurance value	Superior treatment Reduction other cost of healthcare delivery Patient access Preferences Logistics & treatment challenges	Risks Safety Duration & complexity	Outcome Clinical value Cost-effectiveness	Disease rarity Type of disease Disease heterogeneity Other treatment options Disease severity	New treatments Future research Innovation	Patient heterogeneity Knowledge of patient population Personalized medicine

**TABLE 4:** Hierarchy of elements over time.

1991 - 2000	2001–2010	2011–2022
Societal considerations (7)	Cost aspects (22)	Societal considerations (22)
Healthcare process considerations (7)	Economic considerations (14)	Economic considerations (20)
Economic considerations (6)	Drug development complexity (12)	Healthcare process considerations (20)
Effectiveness (4)	Effectiveness (4)	Effectiveness (15)
Cost aspects (3)	Healthcare process considerations (4)	Cost aspects (14)
Innovational aspects (2)	Disease characteristics (3)	Innovational aspects (13)
Disease characteristics (1)	Patient characteristics (2)	Drug development complexity (12)
Patient characteristics (0)	Innovational aspects (2)	Disease characteristics (10)
Drug development complexity (0)	Societal considerations (1)	Patient characteristics (7)

## 4 Discussion

### 4.1 Key findings

The aim of our study was to identify the elements that attribute to value-based pricing of innovative drugs from a pharmaceutical industry’s perspective, in an attempt to resolve the controversy over transparency on drug prices and contribute to a jointly defined and agreed upon framework for value-based pricing as a starting point for value-based contracting.

Reviewing the 31 included publications, we found that all elements that were placed into our framework were covered. Assuming that the emphasis placed or not placed on elements determining the value of innovative drugs was indicated by the number of times these elements appeared in the analyzed publications, our study resulted in the following three key findings.

First, economic considerations and cost aspects associated with the development, registration, manufacturing and marketing of innovative drugs are the two most frequently mentioned elements for establishing a price. Furthermore, innovation, disease characteristics and patient characteristics were least mentioned in relation to value-based pricing. Secondly, effectiveness and, more specifically, cost-effectiveness, being an important parameter of traditional HTA decisions, were hardly mentioned in the reviewed publications. However, healthcare process and societal considerations, likewise important elements of drug value framework preceded cost-effectiveness. And finally, the complexity of drug development should be added as an additional element to drug value.

Regarding the first key finding, our results supported the unexpected importance of economic and cost aspects regarding determination of prices of innovative drugs. Especially considering the pharmaceutical industry’s emphasis on a broader concept of value and their reluctance to cost-based pricing. Gregson’s methodological framework of pricing basics underlined our results. Pricing is a trade-off in which the manufacturer sets the lowest price considering costs and profit and the market sets the upper limit price through a maximum of willingness-to-pay (Gregson et al., 2005b). Nevertheless, in a systematic review by Morgan et al. (Morgan et al., 2011) it was concluded that no ‘golden standard’ was available to estimate the cost of developing a drug. Additionally, Lexchin argued that “drugs are being priced on how desperate patients are, not how much it costs to develop them” (Lexchin, 2017).

Regarding the second key finding, effectiveness and, especially cost-effectiveness, does not qualify as an important element of value-based pricing, except for effectiveness in terms of contributing to outcome, convenience, superiority of the new treatment or reducing other healthcare costs. Moreover, our results demonstrated little or even reverse attention of cost-effectiveness in relation to a drug’s value. Since cost-effectiveness is an important parameter in determining value and, eventually, price in many countries, payers and industry seem miles apart.

This finding was confirmed by several studies in which no relationship was found between price and therapeutic improvement (Suslow, 1992; Vogler and Paterson, 2017). In more recent studies no evidence of a strong relationship was found between effectiveness and the price of orphan drugs or cancer drugs (Onakpoya et al., 2015). However, nowadays, in many countries it is common practice to determine the prices of new innovative drugs, at least partly, based on HTA, cost-effectiveness analysis (CEA) or determination of the incremental cost-effectiveness ratio (ICER) (Vogler et al., 2017). Nevertheless, in the case of the latest cell and gene therapies traditional cost-effectiveness analysis is deemed not appropriate (Coyle et al., 2020).

Simultaneously, pressure on the healthcare system in developed countries is increasing. Time has come that pharmaceutical companies should move forward and should be forced to be more transparent. Shareholders can play an important role and should raise their voices to impose a sustainable and socially responsible business that creates value to all stakeholders.

As mentioned before, in 2020 the EFPIA introduced novel pricing methods that build on effectiveness and outcomes (EFPIA, 2020). Hence, nowadays more attention is given to this element than we found in our research. A recent study by Villa et al. revealed no strong relation between the epidemiology–incidence or prevalence–of rare diseases and their cost of treatment (Villa et al., 2022). Nevertheless, as concluded by Neumann et al*.,* mutual starting points are on valuing the societal and healthcare process benefits of pharmaceuticals (Neumann et al., 2021).

Lastly, regarding drug development complexity, we found that Hughes-Wilson et al. stated that manufacturing complexity, and the level of research undertaken should be part of the evaluation framework of orphan drugs (Hughes-Wilson et al., 2012).

For now, it is important to continue raising awareness on the subject and keep conducting research to quantify and weigh the elements that constitute value. Moreover, it is important that health authorities who establish the maximum price of innovative drug and representatives of pharmaceutical companies agree on which value elements are important to consider in the eventual price. With upcoming one-time treatments in gene and cell therapy it is even more important, because effectiveness and cost-effectiveness may not be sufficient parameters for these treatments in the future, whereas (long term) societal and healthcare benefits may even become more relevant. The same applies to the increasing complexity of drug making of the cell and gene therapies and the complex and personalized ways of drug preparation and administration. Maybe because of these long term and unforeseeable benefits (or risks) and increased complexity a cost-based-plus model might in the end be a solution, whereas the plus can be profit, complexity or any other elements from this review all parties agree upon.

### 4.2 Limitations

Our review has some important limitations. First, it was based on a systematic search of publications in scientific platforms, such as PubMed, Econlit and Embase, and omitted the debate and public statement in publications in more popular magazines, newspapers or pharmaceutical companies’ websites. Furthermore, we did not weigh the elements, instead, we valued them according to the number of times they appeared in journals. Finally, we focused on innovative drugs, thereby not paying attention to value changes over time–i.e., at market introduction, after market entry of competitive alternatives or patent expiry. Furthermore, we considered all elements separately with less attention to possible interlinkages of different elements. For instance, innovation is an element of value, but as mentioned in the introduction paragraph, it is related to, e.g., public health, social and economic needs, or healthcare process convenience considerations. More research is required to prioritize and quantify the weights and dependency of all relevant elements, and to weigh these elements over time. Moreover, it is valuable to search for additional elements of value that have gained more attention currently, such as sustainable production, effectiveness related to gender and fair distribution and availability of drugs.

### 4.3 Conclusion

Although we found similar elements that attribute to the value of innovative pharmaceuticals, both from payers’ or health authorities’ and pharmaceutical industry’s perspectives, finding common ground for agreed upon elements seems very complicated, especially considering the element of (cost-)effectiveness which is an important part of the existing drug value frameworks.

While understandable that cost aspects and economic considerations play an important part in drug pricing, considering the commercial field pharmaceutical companies operate, their prominent presence in publications on the value of innovative drugs was not expected and, therefore, remarkable. Therefore, mutual starting points may be found in the value elements on societal considerations and healthcare process benefits potentially linked to innovation, and the acknowledgement of drug development complexity. Especially because in the last 10 years the order of elements resembles the increasing importance of societal and healthcare process aspects in addition to business considerations.

## Data Availability

The original contributions presented in the study are included in the article/Supplementary Material, further inquiries can be directed to the corresponding author.
