# Supplementary material for: Unraveling elements of value-based pricing from a pharmaceutical industry’s perspective: a scoping review
Source: Front Pharmacol. 2024 Jun 24;15:1298923. doi: 10.3389/fphar.2024.1298923 (PMC11228688; doi:10.3389/fphar.2024.1298923)
Supplement: Supplementary file 1 [file Table1.DOCX]

**S1 File. Search strategy**

**Initial search: February 2020**

| **Used database** | **# total** | **# after deduplication** |
| --- | --- | --- |
| Embase.com (1971-) | 2608 | 2583 |
| Medline ALL ovid (1946-) | 823 | 152 |
| Web of Science Core Collection (1975-) | 776 | 373 |
| EconLit ProQuest (1886-) | 261 | 179 |
| Google Scholar | 200 | 120 |
| **Total** | **4668** | **3407** |

**Updated search strategy: August 2022**

| **Used database** |  |  |
| --- | --- | --- |
| Embase.com (1971-) | 3086 | 3056 |
| Medline ALL ovid (1946-) | 993 | 175 |
| Web of Science Core Collection (1975-) | 1170 | 573 |
| EconLit ProQuest (1886-) | 240 | 130 |
| Google Scholar | 200 | 95 |
| **Total** | **5689** | **4029*** |

*The total number of found publications after the updated search does not correspond with the total number of publications from the first search in February 2020, combined with the uniquely found publications. After linking the publications of the first search (3407 unique publications) to the updated search we found 801 new unique publications. The total number of included publications after finishing both searches is 4208 (3407 + 801). Reasons for the difference may be:

- records were not available anymore in the used databases
- records failed to be selected in the updated search due to less relevance
- Thesaurus terms of records were changed

**Embase.com**

('pricing'/de OR 'value based pricing'/de OR 'drug cost'/de OR 'pharmacoeconomics'/de OR (price OR priced OR prices OR pricing OR drug-cost OR pharmacoeconomic* OR (pharmac* NEAR/3 economic*)):ab,ti) AND ('orphan drug'/de OR 'rare disease'/de OR ('innovation'/de AND 'drug therapy'/de) OR (((orphan OR ultraorphan) NEAR/6 (drug* OR disease* OR designation* OR medicin* OR pharmaceutical* OR product* OR status)) OR ((expensive* OR innovative*) NEXT/1 (drug* OR disease* OR designation* OR medicin* OR pharmaceutical* OR product*)) OR (drug* NEXT/1 (innovation*)) OR ((drug* OR therap* OR pharmac* OR treat*) NEAR/6 (rare OR ultrarare) NEAR/3 (disease* OR disorder*))):ab,ti) NOT ([animals]/lim NOT [humans]/lim)

**Medline ALL ovid**

(Drug Costs / OR Economics, Pharmaceutical / OR (price OR priced OR prices OR pricing OR drug-cost OR pharmacoeconomic* OR (pharmac* ADJ3 economic*)).ab,ti.) AND (Orphan Drug Production/ OR Rare Diseases/ OR (((orphan OR ultraorphan) ADJ6 (drug* OR disease* OR designation* OR medicin* OR pharmaceutical* OR product* OR status)) OR ((expensive* OR innovative*) ADJ (drug* OR disease* OR designation* OR medicin* OR pharmaceutical* OR product*)) OR (drug* ADJ (innovation*)) OR ((drug* OR therap* OR pharmac* OR treat*) ADJ6 (rare OR ultrarare) ADJ3 (disease* OR disorder*))).ab,ti.) NOT (exp animals/ NOT humans/)

**Web of Science Core Collection**

AB=(((price OR priced OR prices OR pricing OR drug-cost OR pharmacoeconomic* OR (pharmac* NEAR/2 economic*))) AND ((((orphan OR ultraorphan) NEAR/5 (drug* OR disease* OR designation* OR medicin* OR pharmaceutical* OR product* OR status)) OR ((expensive* OR innovative*) NEAR/1 (drug* OR disease* OR designation* OR medicin* OR pharmaceutical* OR product*)) OR (drug* NEAR/1 (innovation*)) OR ((drug* OR therap* OR pharmac* OR treat*) NEAR/5 (rare OR ultrarare) NEAR/2 (disease* OR disorder*)))) NOT ((animal* OR rat OR rats OR mouse OR mice OR murine) NOT (human* OR patient*)))

**EconLit ProQues**

((price OR priced OR prices OR pricing OR drug-cost OR pharmacoeconomic* OR (pharmac* N2 economic*))) AND ((((orphan OR ultraorphan) N/5 (drug* OR disease* OR designation* OR medicin* OR pharmaceutical* OR product* OR status)) OR ((expensive* OR innovative*) N/1 (drug* OR disease* OR designation* OR medicin* OR pharmaceutical* OR product*)) OR (drug* N/1 (innovation*)) OR ((drug* OR therap* OR pharmac* OR treat*) N/5 (rare OR ultrarare) N/2 (disease* OR disorder*))))

**Google scholar**

intitle:prices|price|pricing "orphan|rare|expensive|innovative drugs|diseases|designation|medicines|pharmaceutical|products|status"
